# The Longevity Med Summit: insights on healthspan from cell to society

**DOI:** 10.3389/fragi.2024.1417455

**Published:** 2024-07-16

**Authors:** Natalie Falshaw, Michael Sagner, Richard C. Siow

**Affiliations:** ^1^ European Society of Preventive Medicine, Oxford, United Kingdom; ^2^ Ageing Research at King’s (ARK), Faculty of Life Sciences and Medicine, King’s College London, London, United Kingdom; ^3^ Department of Physiology, Anatomy and Genetics, Medical Sciences Division, University of Oxford, Oxford, United Kingdom

**Keywords:** longevity, healthy ageing, healthspan, preventive medicine, public health, biological age, geroscience, diet

## Abstract

In recent years, there has been a paradigm shift with regards to ageing, challenging its traditional perception as an inevitable and natural process. Researchers have collectively identified hallmarks of ageing, nine of which were initially proposed in 2013 and expanded in 2023 to include disabled macroautophagy, chronic inflammation, and dysbiosis, enhancing our understanding of the ageing process at microscopic, cellular, and system-wide levels. Strategies to manipulate these hallmarks present opportunities for slowing, preventing, or reversing age-related diseases, thereby promoting longevity. The interdependence of these hallmarks underscores the necessity of a comprehensive, systems-based approach to address the complex processes contributing to ageing. As a primary risk factor for various diseases, ageing diminishes healthspan, leading to extended periods of compromised health and multiple age-related conditions towards the end of life. The significant gap between healthspan and lifespan holds substantial economic and societal implications. The inaugural Longevity Med Summit (4–5 May 2023, Cascais, Portugal) provided an international forum to discuss the academic and industry landscape of healthy longevity research, preventive medicine and clinical practice to enhance healthspan.

## 1 Introduction

The objective of the inaugural Longevity Med Summit (4–5 May 2023, Cascais, Portugal) was to provide a forum to discuss the current landscape of academic and industry healthy ageing and longevity research, clinical diagnostics, therapeutics, entrepreneurship and innovation. The Summit showcased new insights that may potentially reverse or rectify age-related cellular changes, enhance the management or prevention of age-related diseases, and alleviate the burden of accelerated ageing and the chronic disease epidemic, thereby enhancing healthspan. Healthspan is defined as the period of life spent in optimal health and enjoying a good quality of life, encompassing not just the absence of illness and injury but also overall wellbeing, including physical, mental, and emotional health. By focusing on healthspan, these interventions may inadvertently extend lifespan, as a body in good health, free from chronic disease or disability, functions better and longer. As the gap between healthspan and lifespan narrows, lifespan may be extended, but the primary aim is to improve the quality of life rather than merely increasing its duration.

The speakers provided invaluable insights during the event, centring on five core themes: preventive medicine, diagnostics and therapeutics, cellular and molecular mechanisms, clinical practice, and innovation within the broader ageing and longevity domain. This report presents and discusses these themes, offering commentary on their real-world applications, with the overarching goal of contributing to the realisation of healthy longevity.

## 2 Diagnostics

Longevity diagnostics involve the assessment of key factors related to ageing to interpret and quantify subtle physiological changes that influence healthspan and disease risk. These assessments can predict future health trajectories and inform targeted decision-making regarding lifestyle modifications. Developing ageing biomarkers to monitor the progressive deterioration, or lack of, of cellular and physiological function associated with ageing is an active area of research, and numerous candidate biomarkers have been proposed from molecular changes and image-based characteristics to clinical phenotypes. However, more recently the focus has been on constructing models to predict biological age, to quantify the drift within a particular system from youthful optimal state. These ageing clocks aim to inform and outperform chronological age in predicting health-related outcomes, longevity, and quality of life.

Various ageing clocks have emerged to assess biological age and health status. The first introduced in 2011 and later refined by Steve Horvath was the cytosine phosphate guanine (CpG) islands DNA methylation clock, which incorporates multiple tissues and CpG patterns. Telomere length serves as another indicator of biological age, with shorter telomeres suggesting genomic instability and replicative senescence.

Immunosenescence refers to the changes in the immune system that occur with ageing, affecting both the innate and adaptive immune responses. This process significantly contributes to age-related chronic diseases and multi-morbidity. Often viewed as detrimental, immunosenescence is associated with low-grade, chronic inflammation known as “inflammageing,” and a reduced ability to mount effective antibody and cellular responses against infections and vaccinations. Quantifying immunosenescence and monitoring inflammageing could serve as an “immuno-clock,” offering a measure of biological age.

The glycome, which is the collection of glycoconjugates attached to lipids or proteins, provides insight into the immune system’s state and inflammatory tone (Shkunnikova et al., 2023). Gordan Lauc (University of Zagreb) discussed how the glycome has become another major contender in the ageing clock space, as glycosylation signatures undergo significant changes with age and can serve as functional effectors of inflammageing, contributing to multiple diseases (Memarian et al., 2023). Specifically, monitoring the IgG glycome can offer valuable insights into cardiovascular risk, hypertension, and other health conditions, allowing for personalised medicine approaches before disease onset.

João Araújo (Nova Medical School, Lisbon) highlighted the contribution of gut microbiome composition and function in ageing and its potential as a longevity predictor, although research in this area, particularly regarding biological age from a microbiome perspective, is still in its nascent stages.

Andrea Meier (National University of Singapore) emphasised the importance of body clocks and organ-specific clocks in predicting age-related diseases. Her recent work has revealed that varying rates of ageing across different organ systems form a multiorgan network, where the biological age of one organ can selectively impact another, leading to specific age-related diseases. This understanding enables early identification of individuals at higher risk of ageing-related health issues and informs strategies to potentially mitigate organ-specific ageing in such individuals (Tian et al., 2023).

Evelyne Bischof (Shanghai University) highlighted the pivotal role of Artificial Intelligence (AI) in Longevity Medicine (Marino et al., 2023). AI has facilitated the availability of omics data and the creation of complex learning datasets and models. These advancements empower the development of personalised and preventive protocols, tailored to individual needs, with the aim of restoring biological age. Bischof also discussed the application of Generative AI in longevity medicine, which holds promise for further enhancing biological ageing clocks and deepening our understanding of the ageing process.

All speakers emphasised the complex nature of ageing and the need for a comprehensive approach, rather than relying on a single solution, advocating for a “multiple shots on target” strategy. In the future, integrating multiple different biological clocks and biomarkers that can capture heterogeneous information system wide will enable a precise and more holistic assessment of an individual’s biological age. This multi-modal approach will not only provide a more accurate understanding of ageing but also facilitate the development of personalised and targeted interventions, thus maximising patient outcomes.

## 3 Therapeutics

Translation of academic findings into practical applications within industry and clinical context is of the utmost importance within the field of longevity. The hallmarks of ageing have played a key role in paving the way for providing mechanisms for therapeutics to target and modify them for lowering biological age and enhancing longevity, attracting interest from academia, industry, and investors. Longevity therapeutics may target intracellular, cellular, all the way up to a higher, integrative system-wide level, representing the continuum and multifaceted nature of the ageing process. Several speakers presented various data on the pharmacological and nutraceutical interventions currently under investigation in clinical trials, or in the pipeline, that may contribute to optimal organelle function and signalling pathways, in turn, allowing for healthy ageing.

Nuno Raimundo (University of Coimbra) described how the progressive breakdown of intracellular and molecular pathways are implicated in ageing-related biological networks. Mitochondrial dysfunction, a well-established hallmark of ageing, can adversely affect lysosomal activity, particularly membrane formation, thus disrupting nutrient signalling (Raimundo, 2023). This chronic organelle crosstalk perturbation skews the anabolism-catabolism equilibrium by activating mTOR and inhibiting AMPK, eventually manifesting in age-related disease. Raimundo underscored the potential modifiability of this deterioration and dysregulation through various interventions, presenting novel strategies for enhancing longevity.

Metformin and rapamycin, and its analogues (rapalogues), are among the most extensively studied longevity compounds, targeting AMPK and mTOR, respectively, as discussed by multiple speakers (Sorrenti et al., 2022). Repurposing existing drugs has shown promise in combating ageing. These compounds exhibit a range of beneficial effects, including immunomodulation and alteration of cellular metabolism mechanisms, in both *in vitro* and *in vivo* models of healthy ageing. However, for their prescription solely for longevity purposes, further research is warranted to determine appropriate dosages and potential impacts on other bodily systems.

In addition to repurposing existing drugs, there are ongoing efforts to identify novel compounds and devise effective therapeutic regimes using machine learning and network pharmacology. Longevity and ageing research has necessitated a shift in disease research from reductionist to systems theory. Joao Pedro Magalhaes (University of Birmingham) discussed his research and the emergence of network pharmacology and *in silico* models, incorporating various disciplines such as systems biology, genomics, and proteomics, among others (Vega Magdaleno et al., 2022). These approaches aim to evaluate and validate longevity interventions, predict drug efficacy, potential synergies, and possible side effects, develop targeted therapies for rejuvenation, customise drug treatments for individual patients, and utilise network and machine learning techniques to streamline target and drug discovery, minimising experimental needs while enhancing accuracy and efficiency. By examining drug-network interactions through omics data analysis and network database retrieval, network pharmacology provides comprehensive insights into drug mechanisms and efficacy, making it particularly suitable for investigating longevity therapeutics.

A growing array of supplements, such as NAD+ and its precursors for cellular energy, calorie mimetics for fasting-like effects, and other bioactives and nutraceuticals, are being explored for their potential anti-ageing benefits. These compounds target specific pathways or provide broad antioxidant and anti-inflammatory properties, which can alleviate age-related damage and promote overall health. Despite being promoted by numerous commercial entities, clinicians may exhibit hesitancy due to less stringent regulatory processes. This caution underscores the importance of careful product selection, as contents may not always match label claims. Nonetheless, many of these compounds show promise and merit thorough investigation as effective tools in longevity therapeutics.

At the cellular level, senolytics are a class of drugs that aim to interfere with senescent cells. Several speakers outlined the process of cellular senescence and its contribution to unhealthy ageing via mechanisms including disrupting tissue functionality and limiting the regenerative potential of adult stem cells. The accumulation of senescent cells leads to an increase in biological age and increases the risk of disease. Senolytics hold promise in eliminating senescent cells, and in model organisms, they have shown potential to extend lifespan, enhance healthspan, and treat or even reverse age-related diseases.

Stem cell exhaustion, another hallmark of ageing, may be addressed through autologous stem cell transplantation, as outlined by Elena Rusyn (American Cell Technology, United States). During the ageing process, the reserves of stem cells become depleted, leading to a reduced regenerative capacity, however by harnessing the body’s own regenerative potential, autologous stem cells may contribute to tissue repair and regeneration, and the complication of possible rejection, as is the case with allogeneic treatments is eliminated.

Advancing to the systemic level, dysbiosis and chronic inflammation, recently recognised hallmarks of ageing, are interconnected and responsive to dietary interventions, as elucidated by Richard Siow (King’s College London). Bioactive compounds in whole foods, potentially acting synergistically to mitigate age-related changes and enhance vitality pathways, are currently being investigated by commercial entities to isolate and validate them *in vivo*. Siow also emphasised the significant impact of dietary choices on rates of functional decline. With the emergence of longevity nutrition frontiers such as nutrigenomics, personalised nutritional regimens tailored to individuals’ genetic profiles are now feasible, enhancing the effectiveness of interventions.

In longevity clinical medicine, therapeutics extending beyond drugs and supplements encompass various approaches aimed at promoting healthy ageing and extending lifespan at a whole-body level. Shai Efrati (Tel Aviv University) presented data on his work with hyperbaric medicine, offering a promising strategy to address oxygen deficiency associated with degenerative ageing and promote regenerative processes. Hyperbaric oxygen therapy (HBOT) has shown potential in supporting neurogenesis, extending telomeres, and reducing senescent cells (Hachmo et al., 2020). Efrati emphasised that it is critical to recognise that the efficacy of HBOT chambers lies in the hyperoxic-hypoxic paradox, where it is the change in pressure, and therefore, oxygen saturation rather than the absolute oxygen level that influences the body’s perceived oxygen levels and elicits positive effects. Commercial efforts to provide more affordable alternatives that overlook the necessary pressure change may not effectively promote healthy ageing.

To promote longevity and lower biological age, pharmaceuticals, nutraceutical supplements, and other interventions should be considered as part of a comprehensive anti-ageing regimen that incorporates other complementary protocols. These may include adopting a healthful diet, engaging in regular exercise and resistance training, practising intermittent fasting, managing stress effectively, and optimising sleep patterns.

## 4 Mechanisms of ageing

Longevity research is a dynamic field, continually generating new hypotheses and pertinent findings. From investigations spanning the brain, gut, cellular milieu, to multi-generational effects, our comprehension of ageing progresses swiftly, revealing the vast expanse of our yet-unexplored knowledge. Alongside diagnostic and therapeutic advancements, several speakers at the Longevity Med Summit disseminated their research findings within their respective domains.

Cláudia Cavadas (University of Coimbra) highlighted the crucial role of the hypothalamus in ageing and energy balance, specifically its regulation of inflammation to mitigate systemic ageing. By studying hypothalamic signalling molecules such as neuropeptide Y (NPY) and ghrelin (Ferreira-Marques et al., 2023), which mimic the neurochemical response to caloric restriction and fasting, researchers have discovered their potential to directly delay ageing by targeting multiple hallmarks. This opens up promising therapeutic avenues for mitigating age-related decline. Furthermore, disruptions in circadian rhythm were shown to accelerate ageing (Carvalhas-Almeida et al., 2023) through hypothalamic involvement, but interventions aimed at restoring the complex neural circuitry involved in metabolic, circadian, and sleep disorders may improve biological clock disruptions. Understanding the hypothalamus, neuropeptides, and sleep-related factors provides insights into delaying age-related decline and enhancing overall health and longevity.

Aubrey de Grey (LEV Foundation, United States) presented the concept of rejuvenation biotechnology, which seeks to repair age-related damage through LEV Foundation’s flagship research program. By implementing interventions which have previously demonstrated efficacy, that target multiple aspects of damage, including rapamycin, senolytic compounds, telomerase gene therapy, and haemopoietic stem cell transplantation, it may be possible to extend healthspan, lifespan, and potentially achieve robust rejuvenation and the restoration of youthfulness in C57Bl/6J mice who have already experienced moderate ageing. The focus is not just on increasing lifespan but also on improving the quality of life during the extended period.

Paulo Oliveira (University of Coimbra) highlighted how mitochondrial dysfunction is a hallmark of ageing, often indicated by damaged mitochondrial DNA, impaired mitophagy, and oxidative stress causing inflammageing. Oliveira presented preliminary data on the effect of maternal nutrient restriction during pregnancy and its impact the ageing trajectory of offspring, through *in utero* mitochondrial programming, potentially leading to accelerated ageing in the brain and heart, as well as increased risk of cardiometabolic and kidney diseases (Pereira et al., 2023).

## 5 Clinical longevity

With the advent of novel diagnostic tools and an expanded array of therapeutic agents aimed at extending healthspan, several commercial longevity clinics have emerged. While mainly positioned as “luxury hospitality and medical services,” these organisations possess a wealth of valuable untapped data from clients from whom have benefited from longevity focused therapeutic interventions. Several speakers emphasised the importance of standardising and validating data collection methods across these clinics by an independent entity. This data could be utilised by researchers and other stakeholders in the field to foster collaboration and advance the knowledge base of longevity research. However, it is important to note that the field of longevity is still emerging, allowing room for experimentation. Consequently, these programmes are often unregulated and come with a substantial price tag.

In addition to these clinics providing to the wealthy to elevate health status, democratising extension of healthy lifespan and applying longevity science to developing preventive measures against age-related disease is of critical importance. For example, Tzipi Strauss (Sheba Medical Centre, Israel) presented the Health Longevity Roadmap for the Sheba Longevity Centre, a pioneering longevity unit in a major public hospital. Recognising longevity as a crucial field encourages clinicians to adopt a proactive approach in helping patients maintain and improve their health, rather than relying solely on reactive treatments for managing diseases.

Advancing longevity medicine into mainstream clinical practice requires educating physicians (Bischof et al., 2021) and integrating longevity principles into medical school curricula, facilitating the essential paradigm shift in healthcare. Michael Sagner (European Society of Preventive Medicine, United Kingdom) introduced the concept of “P4 medicine,” highlighting the importance of transitioning clinical care towards being predictive, preventive, personalised, and participatory (Sagner et al., 2017). This approach holds promise in addressing the rising burden of slowly progressing, “silent” age-related chronic diseases prevalent in modern society through lifestyle and preventive medicine strategies.

## 6 Innovation in the broader longevity domain

Longevity research, also referred to as geroscience (Larsen, 2017), employs a multidisciplinary scientific approach to understanding the mechanisms of ageing, identifying potential interventions, and developing novel strategies to promote longevity and delay, prevent, or reverse age-related disease. The summit provided a unique platform not only for scientists and physicians in longevity research but also for industry experts and entrepreneurs who are driving innovation in this field. These individuals had the opportunity to showcase their current products and share insights on upcoming developments within funding research, technology, and multi-stakeholder collaboration, further enriching the diversity of perspectives and contributions to the advancement of longevity research.

For instance, leveraging Web3 and blockchain technologies to decentralise the research ecosystem may alleviate the problem of “stagnant science.” Decentralised autonomous organisations (DAOs), offering improved incentives and funding avenues, empower collective intelligence to advance scientific discovery, disrupting the traditional centralised funding model. These innovative solutions, especially in the realm of longevity, aim to enhance the speed, fairness, and transparency of scientific research and publishing (Oka et al., 2023).

The field of longevity offers fertile ground for innovative approaches to scientific research because it inherently embodies the convergence of biology and technology. Longevity research naturally integrates advancements in healthcare technology to propel progress in this domain. By harnessing the power of omics data analysis, researchers can capture and analyse vast amounts of biological information, providing insights into longevity-related factors at multiple levels of complexity.

Moreover, hyper-personalisation will be pivotal in the development of anti-ageing solutions, offering a deeply customised and individualised approach to healthcare. The integration of biomarker and wearable data was explored, with health-tech emerging as a cornerstone of this personalised strategy, enabling healthcare providers to access real-time insights into an individual’s health and wellbeing.

This transitioned into discussions on how a significant portion of healthcare data resides outside of healthcare facilities, highlighting the pivotal role the cloud will play in storing and accessing this vast reservoir of information in the future. Emphasis was placed not only on data collection but also on the decision-making processes fuelled by this data. Healthcare automation encompasses tasks such as data aggregation, normalisation, analytics, and engagement, which will be necessary to optimise the use of available information.

To address data safety concerns, blockchain technology can provide an additional layer of security and integrity for healthcare data. By leveraging these technological advancements and adopting a multidisciplinary approach, the field of longevity research is poised to accelerate its progress, unlocking new insights and potential solutions with greater efficiency and accuracy.

Commentaries surrounding the longevity funding ecosystem, technology integration, and longevity data considerations shifted towards the challenges inherent in healthcare systems themselves. There was a call to reimagine the response to the escalating burden of chronic diseases at individual, societal, and global levels, aiming to enhance access to healthier, longer lives for all. Tina Woods introduced the concept of the exposome, which refers to the sum total of exposures that influence human health. By addressing these interacting lifestyle factors, such as diet and physical activity; socioeconomic determinants, such as discrimination, early and lifelong education, training and skills, financial status, and social support; and, increasingly, the characteristics of physical environments, such as green spaces and air quality, we can mitigate systemic chronic inflammation and promote lifelong health (Woods et al., 2022). This comprehensive strategy necessitates the integration of science, technology, policy, regulatory reforms, public engagement, and private sector investment to advance the mission of healthy longevity. Discussions also explored how the United Kingdom could capitalise on Brexit to enhance citizen wellbeing by creating open databases and platforms for testing interventions and sharing data, positioning itself as a global leader in health innovation.

## 7 Limitations in longevity research and practice

The field of longevity medicine faces several limitations that hinder its progress and acceptance within the mainstream medical community. These limitations include:

1. Uncertainty in ageing classification. There is no consensus on when age-related functional decline becomes pathological, making it challenging to acquire funding and grants for longevity research. The classification of ageing as a disease or a natural process remains uncertain.
2. Time constraints in clinical studies. Clinical studies cannot feasibly have lifespan as a primary outcome due to the extended time required to observe the effects. Therefore, surrogate biomarkers of lifespan or health span are necessary to advance human trials in longevity research.
3. Biomedical research and clinical practices. Current biomedical research relies on statistical correlations and population averages, while clinical practices focus on disease treatment rather than health maintenance. Shifting away from the standard “one-size-fits-all” approach and towards personalised medicine accounting for individual variation is crucial, as no one is a true average.
4. Resistance to recognising ageing as a disease. There is a general resistance to recognising ageing as a disease, often due to discomfort associated with the idea of slowing the course of ageing or hinting at the possibility of immortality. Many perceive ageing as a natural part of life and view departing from this cycle as a violation. However, frailty should not be considered a requisite for a meaningful life, and suffering at the end of life should not be accepted as inevitable.
5. Low evidence levels and slow development. The slow development of the field has led to low levels of evidence for longevity-based interventions. As a nascent field, longevity research faces challenges in gathering robust evidence, which further hinders its acceptance within the mainstream medical community.

**FIGURE 1:**
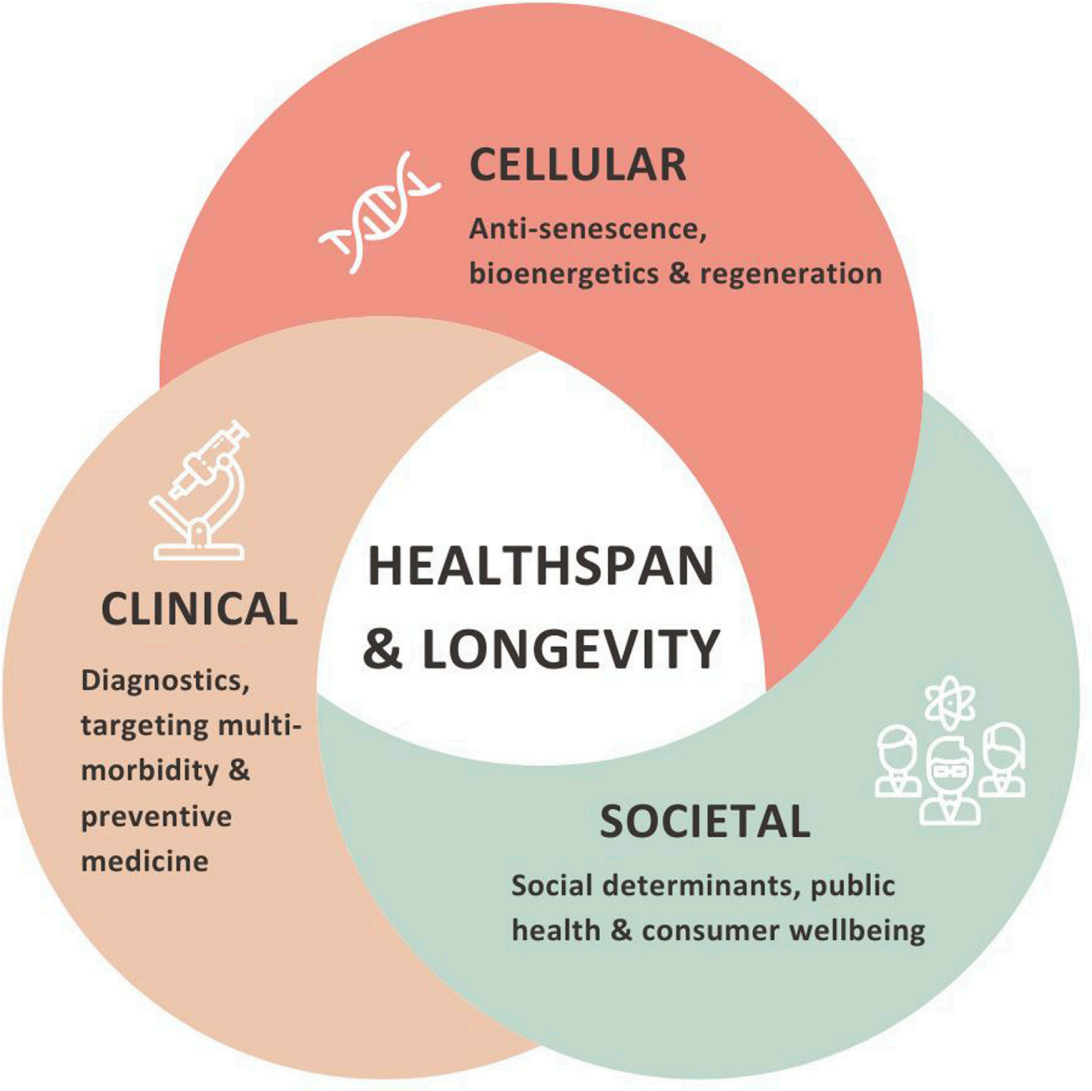
To effectively tackle the causes and consequences of ageing, mitigating age-related decline, and enhancing healthspan and longevity, public health responses must be comprehensive and multi-disciplinary, spanning from cellular mechanisms to clinical applications and community solutions. This approach should consider the physiological aspects (cellular mechanisms and clinical translation), clinical interventions (enhanced tecnologies and access to clinical delivery), and societal implications (lifestyle and social interventions for ageing populations to mitigate age-related decline and promote healthy longevity).

Addressing these limitations and fostering a deeper understanding of the ageing process can pave the way for advancements in longevity research and the development of effective interventions to promote healthy ageing and extend human lifespan (Figure 1).

## 8 Conclusion

The Longevity Med Summit showcased the active and dynamic nature of the field of longevity. The traditional view of ageing as an inevitable process is being challenged, with a focus on redefining it as a treatable condition. Ageing is a primary risk factor for numerous diseases, and narrowing the gap between healthspan and lifespan and preventing or delaying the onset of age-related disease was deemed a priority. The summit provided insights into significant developments in the practical aspects of treating ageing (clinical), advancing scientific understanding of ageing (research), and promoting creative solutions in the pursuit of longer and healthier lives (innovation), from repurposing existing drugs to exploring new scientific findings and emerging technologies. Despite limitations and challenges, the field of longevity medicine is paving the way for experimentation, innovation, and potential collaborations, making it an exciting area with the potential to improve health outcomes and extend lifespan for individuals seeking to age in a healthier and more fulfilling manner.
